# Microbial biotransformation of *Pericarpium Citri Reticulatae* (PCR) by *Aspergillus niger* and effects on antioxidant activity

**DOI:** 10.1002/fsn3.2049

**Published:** 2020-12-04

**Authors:** Fu Wang, Lin Chen, Shiwei Chen, Hongping Chen, Youping Liu

**Affiliations:** ^1^ Department of Pharmacy Standardization Education Ministry Key Laboratory of Traditional Chinese Medicine Chengdu University of TCM Chengdu China; ^2^ Food & Drugs Authority of Nanchong Nanchong China

**Keywords:** antioxidant activity, *Aspergillus niger*, Microbial transformation, *Pericarpium Citri Reticulatae*, UPLC‐ESI‐MS/MS

## Abstract

*Pericarpium Citri Reticulatae* (PCR), the mature fruit peel of *Citrus reticulata Blanco* and its different cultivars, is an important citrus by‐product with beneficial health and nutritive properties. However, due to the lack of value‐added methods for its development and utilization, a large amount of PCR is discarded or wasted. To explore a possibly more effective method to utilize PCR, we compared the chemical and biological differences before (CK) and after (CP) microbial transformation of PCR by *Aspergillus niger*. UPLC‐ESI‐MS/MS, HPLC, and LC‐MS methods were used to compare the chemical profiles of CK and CP. The results demonstrated that microbial biotransformation by *A. niger* could transform flavonoid compounds by utilizing the carbohydrate and amino acid nutrients in PCR. This could also promote the accumulation of polyhydroxyflavones compounds in CP. The antioxidant assay demonstrated that CP had significantly greater free radical‐scavenging activity than CK. The higher antioxidant activity of CP may result from the high level of flavonoids with associated phenolic hydroxyl groups. Microbial biotransformation is an effective method for improving the antioxidant capacity of PCR and may be effective and useful in other natural product situations.

## Graphical abstract

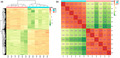

## INTRODUCTION

*Pericarpium Citri Reticulatae* (PCR), the mature fruit peel of *Citrus reticulata Blanco* and its different cultivars, is an important citrus by‐product used by the pharmaceutical and food industries (Yu, Sun, et al., 2018). Phytochemical investigations indicated that flavonoids, volatile components, and alkaloids are the major bioactive components of PCR (Yu, Zhang, et al., 2018). Because of its unique aroma and health benefits, it is popular and used as a food, tea, and seasoning. However, due to the lack of high value‐added methods for its development and utilization, a large amount of PCR is discarded or wasted.

Microbial biotransformation of flavonoids has been studied because of the diversity of the enzyme systems, easy production of high‐value products, and high selectivity. The processes involved are environmentally friendly and nonpolluting (Cao et al., 2015; Kumar & Pandey, 2013). Flavonoids are common plant secondary metabolites that have been developed into many food, medicine, and health products (Gonzales et al., 2015). Flavonoids have a variety of pharmacological activities, anti‐inflammatory (Gil‐Cardoso et al., 2016), antioxidation (Jia et al., 2012), antithrombosis (Chen et al., 2012), antitumor (Jiang, Zhu, et al., 2019), antithrombosis (Vazhappilly et al., 2019), antibacterial (Xie et al., 2015), antiviral (Zakaryan et al., 2017), hypolipidemic (Bao et al., 2016), antituberculosis (Tao et al., 2019), antiatherogenic (Basu et al., 2016), anti‐Alzheimer's disease (Shahinozzaman et al., 2018), and liver protection (Jiang, Yan, et al., 2019). *Aspergillus niger* is a fungus commonly used in the microbial biotransformation of chemical components (Kang et al., 2019). Consumption of *A. niger* is safe, and it is widely used in the food industry (Schuster et al., 2002). *Aspergillus niger* has also been used in the biotransformation of flavonoids such as flavone (Parshikov and Sutherland, 2015), flavonol (Kostrzewa‐Susłow et al., 2014), flavonoid glycoside (Cao et al., 2015), flavanone (Kostrzewa‐Susłow and Janeczko, 2012), polymethoxy flavonoids (Sanchez‐Gonzalez & Rosazza, 2004 ), chalcone, and isoflavanone (Abdella et al., 2018). The biotransformation mechanism may involve the formation of transformation products through hydroxylation, methylation, dehydrogenation, and other processes with the participation of enzymes produced by *A. niger* (Caspani et al., 2019; Bianchini et al., 2015). However, little is known about the specific enzymes involved in the biotransformation. The same *A. niger* strain may produce different conversion products in response to different flavonoids, indicating that the microbial transformation can be highly specific (Das & Rosazza, 2006). Present research involves screening the metabolic enzymes of *A. niger* and the biotransformation of chemical components.

*Pericarpium Citri Reticulatae* contains large amounts of flavonoids, with hesperidin was identified as the major compound. To date, only a small number of constituents, such as naringenin (Xu et al., 2012), rutin (You et al., 2010), tangeretin (Mahmoud et al., 2008), and nobiletin (Okuno & Miyazawa, 2004), have been reported to be transformed by *A. niger*. No studies have analyzed the chemical profiles and the biological differences before and after microbial biotransformation of PCR by *A. niger*.

We selected PCR as the experimental material and transformed it with a strain of *A. niger*. We compared the chemical and biological differences before (CK) and after (CP) microbial transformation by *A. niger*. UPLC‐ESI‐MS/MS techniques were used to compare the chemical profiles of CP and CK. HPLC coupled with diode array detector (HPLC–DAD) method and LC‐MS were used to determine the major constituents in CP and CK. Three different methods (DPPH, FRAP, and ABTS) were used to evaluate the antioxidant activity. The objective of this study was to provide a practical method for the additional development and use of PCR.

## MATERIALS AND METHODS

### Microbial transformation

The strain of *A. niger* (3.13901) was isolated from soil and preserved in the China General Microbiological Culture Collection Center. The methods refer to the reported literature (Stankov‐Jovanović et al., 2015). The details are as follows: an 8 g sample was weighed and spread in a petri dish. It was sterilized by ultraviolet irradiation on an ultra‐clean workbench for 30 min, then turned over, and sterilized by irradiation for an additional 30 min. The sterilized samples were divided into the reverse inoculation group (CP) and the control group (CK). The spore suspension was obtained by eluting *A. niger* culture dish with sterile normal saline and filtered with absorbent cotton; then, 1 ml spore suspension was diluted 1,000 times with normal saline to obtain the standard spore suspension. CP: 1 ml standard *A. niger* spore suspension (10^6^ cfu/ml) was added to each petri dish (*n* = 6). CK: We added 1 ml of sterile water per petri dish (*n* = 6). The two groups of samples were cultured in an artificial climate chamber at 30°C with 95% RH. Samples were removed for detection after 5 days.

### Metabolites extraction

A 50 mg sample was added to an EP tube and 1,000 μl of extraction solution (acetonitrile: methanol: water = 2:2: 1) containing internal standard (L‐2‐chlorophenylalanine, 2 μg/ml) was added. After a 30 s vortex, the samples were homogenized at 35 Hz for 4 min and sonicated for 5 min in an ice‐water bath. The homogenization and sonication cycle was repeated two times. Then the samples were incubated at −40°C for 1 hr and centrifuged at 11180  *g* for 15 min at 4°C. A 250 μl sample of the supernatant was transferred to a fresh tube and dried in a vacuum concentrator at 37°C. The dried samples were reconstituted in 400 μl of 50% acetonitrile by sonication on ice for 10 min. The solution was then centrifuged at 18894.2  *g* for 15 min at 4°C, and 75 μl of the supernatant was transferred to a fresh glass vial for LC/MS analysis. The quality control (QC) sample was prepared by mixing an equal aliquot of the supernatants from all of the samples (Wu, Jiao, et al., 2018).

### LC‐MS/MS analysis

UHPLC separation was carried out using a 1,290 Infinity series UHPLC System (Agilent Technologies), equipped with a UPLC BEH Amide column (2.1 × 100 mm, 1.7 μm, Waters). The mobile phase consisted of 25 mmol/L ammonium acetate and 25 mmol/L ammonia hydroxide in water (pH = 9.75) (A) and acetonitrile (B). The analysis was conducted with an elution gradient as follows: 0 ~ 0.5 min, 95% B; 0.5–7.0 min, 95%–65% B; 7.0 ~ 8.0 min, 65%–40% B; 8.0–9.0 min, 40% B; 9.0–9.1 min, 40%–95% B; and 9.1–12.0 min, 95% B. The column temperature was 25°C. The auto‐sampler temperature was 4°C, and the injection volume was 1 μL (pos) or 1 μL (neg), respectively. The TripleTOF 6,600 mass spectrometry (AB Sciex) was used for its ability to acquire MS/MS spectra on an information‐dependent basis (IDA) during an LC/MS experiment. In this mode, the acquisition software (Analyst TF 1.7, AB Sciex) continuously evaluates the full scan survey MS data as it collects and triggers the acquisition of MS/MS spectra depending on preselected criteria. In each cycle, the most intensive 12 precursor ions with intensity above 100 were chosen for MS/MS at collision energy (CE) of 30 eV. The cycle time was 0.56 s. ESI source conditions were set as following: Gas 1 at 60 psi, Gas 2 at 60 psi, Curtain Gas at 35 psi, Source Temperature at 600°C, Declustering potential at 60 V, and Ion Spray Voltage Floating (ISVF) at 5,000 V in positive mode (Shimizu et al., 2018).

### Data preprocessing and annotation

MS raw data (.wiff) files were converted to the mzXML format by ProteoWizard and processed by R package XCMS (version 3.2). The process included peak deconvolution, alignment, and integration. Minfrac and cutoff were set as 0.5 and 0.3, respectively. In‐house MS2 database was used for metabolite identification.

### Determination of total antioxidant capacity

Approximately 2.0 g of citrus peels from each sample were freeze‐dried using a vacuum freeze dryer and then ground into powder using a mortar. The antioxidant activities of citrus peels were evaluated by the DPPH radical scavenging activity assay. Briefly, 0.02 g of citrus peels was mixed with 180 µl of a DPPH working solution. The mixture was incubated at room temperature for 30 min in darkness. The absorbance was measured at 517 nm with a microplate reader (Sirivibulkovit et al., 2018). The ABTS radical scavenging capacities of citrus peels were conducted with a Total Antioxidant Capacity Assay Kit with ABTS method (Beyotime Biotechnology Co., Ltd.). Trolox was used as a standard compound. A calibration curve was prepared with different concentrations of Trolox in solution, and the results were expressed as mmol TEAC/L of citrus peels where TEAC is defined as the Trolox equivalent antioxidant capacity (Polak & Bartoszik, 2018). The reducing abilities of citrus peels were measured by a Total Antioxidant Capacity Assay Kit with the FRAP method (Beyotime Biotechnology Co., Ltd., Shanghai, China). The standard curve was constructed using FeSO4 solution, and the results were expressed as l M Fe(II)/g dry weight of the citrus peels (Mozaffari et al., 2018).

### Targeted verification of hesperidin and vitex

Hesperidin and vitex were analyzed as described previously with some modifications (Miura et al., 2020). A 200 mg sample of powder was weighed and extracted overnight at 4°C in 1.0 ml of 70% aqueous methanol. Following centrifugation at 10,000 × *g* for 10 min, the extracts were absorbed (CNWBOND Carbon‐GCB SPE Cartridge, 250 mg, 3 ml; ANPEL) and filtered (SCAA‐104, 0.22 µm pore size; ANPEL) before LC‐MS analysis. The HPLC conditions were as follows: HPLC: column, Waters ACQUITY UPLC HSS T3 C18 (1.8 µm, 2.1 mm × 100 mm), solvent system, water: acetonitrile, gradient program, 90:10 v/v at 0 min, 90:10 v/v at 1.0 min, 10:90 v/v at 3 min, 10:90 v/v at 5 min, 10:90 v/v at 6 min; flow rate, 0.42 ml/min; temperature, 40°C; and injection volume: 2 µl. The TripleTOF 6,600 mass spectrometry (AB Sciex) was used for its ability to acquire MS/MS spectra on an information‐dependent basis (IDA) during an LC/MS experiment. The ESI source operation parameters were as follows: ion source, turbo spray; source temperature 500°C; ion spray voltage (IS) 5,500 V; ion source gas I (GSI), gas II (GSII), and curtain gas (CUR) were set at 55, 60, and 25.0 psi, respectively.

### Targeted verification of quentin

Quentin was analyzed by HPLC, as described previously with some modifications (Xiao et al., 2020). The mobile phase, a mixture of buffer (0.4% phosphoric acid), and methanol (50:50 v/v) were filtered through a 0.45 µm membrane filter and degassed by sonication. HPLC analysis was performed at 30°C with a flow rate of 0.5 ml/min, and the samples were injected into an ODS C18 (4.6 mm × 250 mm) column (Beckman Coulter Inc.). The column effluent was monitored at 360 nm. Quantification was performed by comparing the peak areas obtained from the samples with those of standards.

### Targeted verification of narirutin and naringenin

Narirutin and naringenin were determined according to the method described by Wang (Wang et al., 2018). We used 0.2 g powder and added 25 ml of methanol. The mixture was refluxed for 1 hr in a 75°C water bath. Following centrifugation at 10,000 × g for 5 min, the extracts were absorbed (CNWBOND Carbon‐GCB SPE Cartridge, 250 mg, 3 ml; ANPEL, Shanghai, China) and filtrated (SCAA‐104, 0.22 µm pore size; ANPEL, Shanghai, China) before use. Chromatographic Conditions were as follows: chromatographic column was a Hypersil BDS C18 (4.6 × 200 mm, 5 μm); solvent system: 0.05% phosphoric acid water and acetonitrile. The gradient elution program was time: 0–5 min, acetonitrile: 20%–25%; time: 5–10 min, acetonitrile: 25%–30%; time: 10–20 min, acetonitrile: 30%–50%; time: 20–30 min, acetonitrile: 50%–60%; time: 30–35 min, acetonitrile: 60%–90%; time: 35–40 min, acetonitrile: 90%–100%; column temperature: 30°C; flow rate: 0.7 ml/min; wavelength: 283 nm and 335 nm; injection volume: 5.0 µl.

## RESULTS

### Metabolic profiling

**FIGURE 1:**
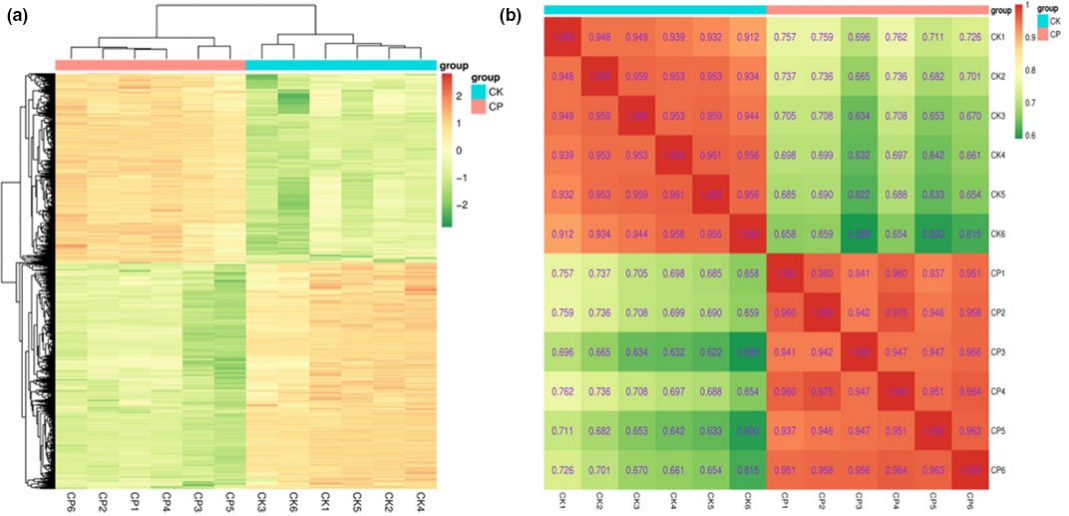
(a) Clustering heat‐map of metabolites between *Pericarpium Citri Reticulatae* (CK) and *Pericarpium Citri Reticulatae* (CP). The upregulated and downregulated metabolites were expressed with different shade colors of red and green, respectively. With the increase in the abundance value, the color of the bar changed from green to red. When the abundance value was 0, the color of bar was white, as shown in the bar at the upper right. (b) Correlation between *Pericarpium Citri Reticulatae* (CK) and *Pericarpium Citri Reticulatae* (CP). The correlation analysis between samples was used to estimate the biological duplication among samples within a group. The closer *R* ^2^ is to 1, the stronger the correlation between the two repeated samples

The metabolites of the citrus peels from CK and CP were investigated based on UPLC‐ESI‐MS/MS and relevant databases. In this study, 2,136 metabolites were detected (Table S1), and 767 of the metabolites were identified (Table S2). All of the metabolites were putatively identified according to the retention time, accurate mass, MS2 and searches of the Human Metabolome Database (HMDB), MassBank, a comprehensive species‐metabolite relationship database (KNAPSAcK), and LIPID MAPS Structure Database (LMSD) metabolomics databases. A heat‐map was applied to visualize the variations of the different metabolites in CK and CP (Figure 1a). The content of metabolites in CP greatly contrasted with that of the CK. This showed that the metabolic transformation caused by *A. niger* had substantial effects on the metabolic components in the citrus peels. Figure 1b shows that the correlation coefficient *R* ^2^ within the group were all greater than 0.9, indicating good repeatability between samples. This finding was demonstrated by clustering analysis of the two samples and showed that they could be clearly distinguished from each other.

### PCA and OPLS‐DA analyses of differential metabolites

**FIGURE 2:**
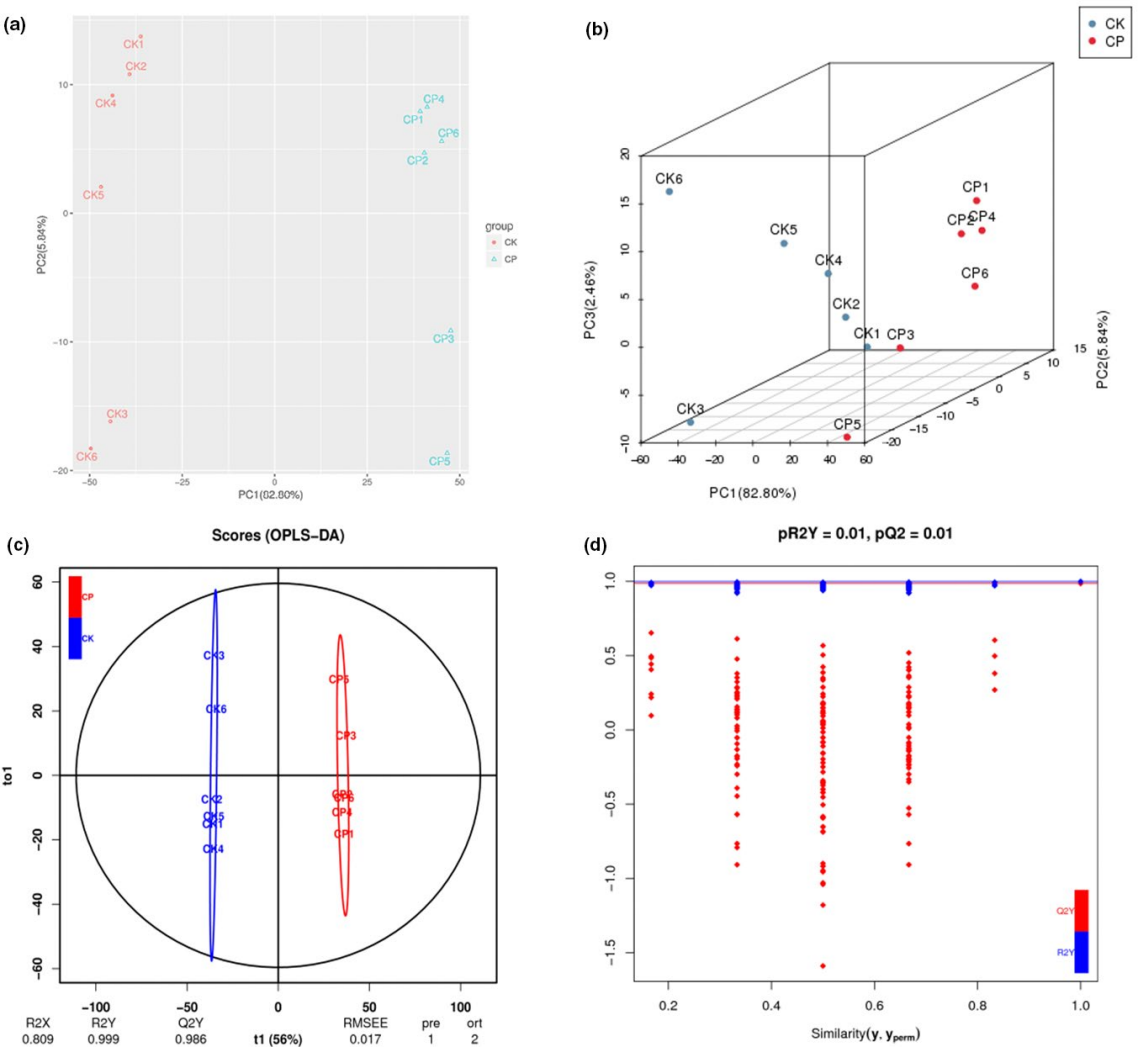
(a) Differential metabolites analysis on the basis of principal component analysis (PCA). (b) PCA 3D plots. (c) Orthogonal signal correction and partial least squares‐discriminant analysis (OPLS‐DA). (d) Validation plots of OPLS‐DA model. The horizontal lines correspond to *R* ^2^ and *Q* ^2^ of the original model, and the blue and red dots represent *R* ^2^′ and *Q* ^2^′ of the model after Y substitution, respectively. If *R* ^2^′ and *Q* ^2^′ are both smaller than *R* ^2^ and *Q* ^2^ of the original model, (the corresponding points do not exceed the corresponding lines), it indicates that the model is of significance. Differential metabolites can be analyzed and screened according to Variable Importance in the Projection (VIP)

Principal component analysis is an unsupervised pattern recognition method used for analyzing, classifying, and reducing the dimensionality of numerical datasets in multivariate problems (Ardila et al., 2015). This approach has been widely used for quality control of herbal medicines. Similarly, OPLS‐DA analysis maximizes the variations between groups and is commonly used to screen differential metabolites (Triba et al., 2015). In this study, PCA was carried out to provide additional insight into the chemical differences between CK and CP. As shown in Figure 2a, the cumulative contribution rate of PC1 and PC2 was 88.64%, with 82.80% attributed to PC1 and 5.84% attributed to PC2 (Figure 2a,b). The classification results of PCA show noticeable differences between the CP and CK. Of the differential metabolites, they were used to establish an OPLS‐DA model. The parameters of log2FC, *p*‐value, and VIP values are shown in Table S3. The results presented in Figure 2c demonstrate that the *R* ^2^ *X*, *R* ^2^ *Y*, and *Q* ^2^ values determined using this model are 0.809, 0.999, and 0.986, respectively. Considering that *Q* ^2^ exceeds 0.9 and the red and green dot did not exceed the corresponding line (Figure 2d), the OPLS‐DA model is stable and reliable and it can be used to identify the differential metabolites.

### Differential metabolite screening analysis

**FIGURE 3:**
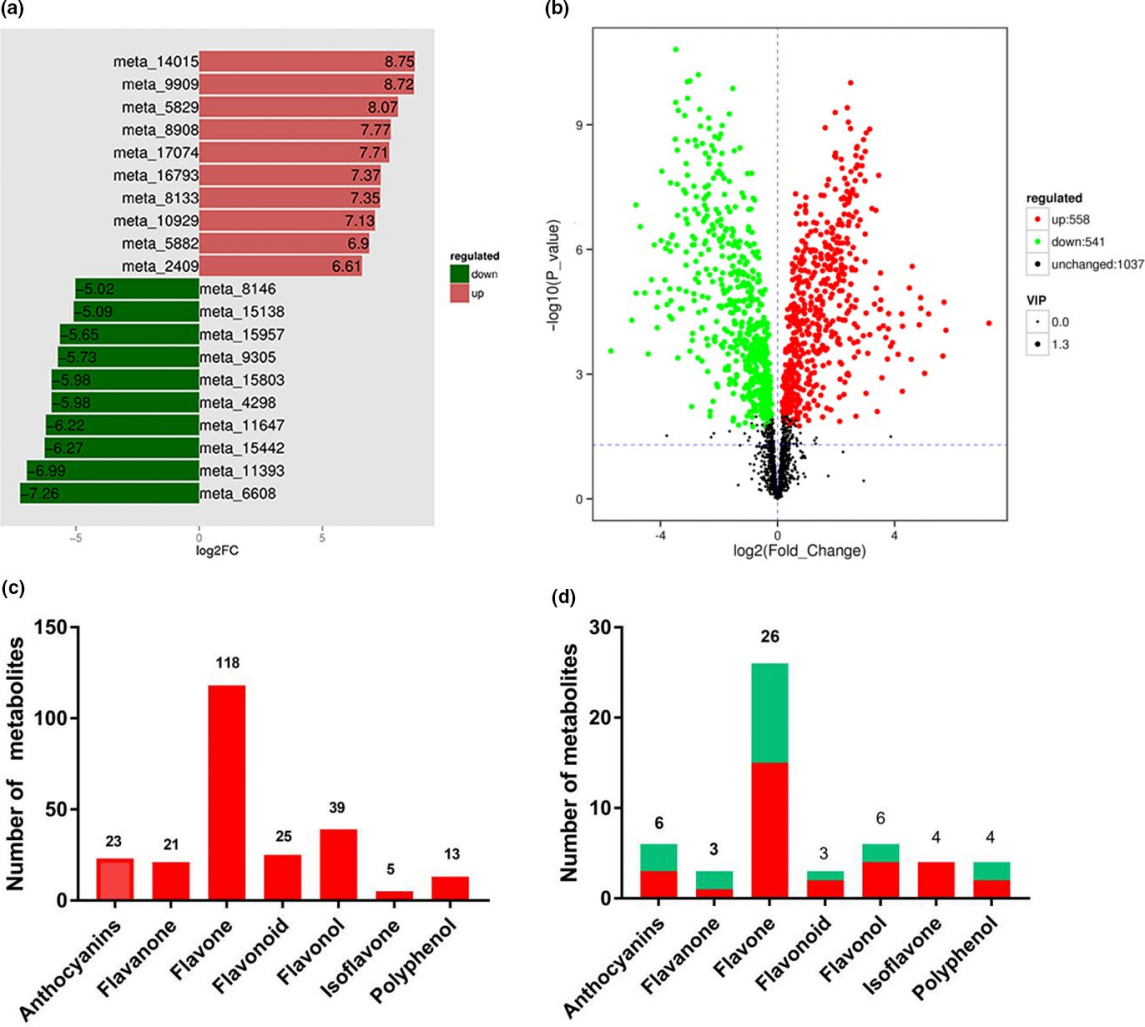
Differential metabolites analysis. (a) Volcano plots of different metabolites. The green dots in the figure represent downregulated differential metabolites, the red dots represent upregulated differential metabolites, and the black dots represent metabolites detected but that are not significantly different. (b) Histogram of fold change. The green pillars in the figure represent downregulated differential metabolites, the red pillars represent upregulated differential metabolites. (c) Number of different types of detected flavonoids metabolites. (d) Number of different types of differential flavonoids metabolites. Red bars represent upregulated differential flavonoids metabolites; green bars represent downregulated differential flavonoids metabolites

The differential metabolites of CK and CP were screened based on the fold change and variable importance in project (VIP) values of the OPLS‐DA model. Specifically, the metabolites having fold change values ≥2 or ≤0.5 and VIP values ≥1 were identified as differential (Table S3). The fold change of metabolites of the two samples was compared and analyzed, and the metabolites with greater changes are shown in Figure 3a after log2 treatment. The volcano plots of different metabolites (Figure 3b) show that the number of compounds upregulated and downregulated was 558 and 541, respectively. The number of compounds remaining unchanged was 1,037. From these large datasets, we focused on the changes of flavonoids because of the metabolic transformation of flavonoids by *A. niger*. Figure 3c shows that 244 flavonoid metabolites were identified, including 118 flavones, 39 flavonols, 25 flavonoids, 21 flavanones, 23 anthocyanins, 5 isoflavones, and 13 polyphenols. There were 52 significantly different flavonoid metabolites between CP and CK (Figure 3d), including 26 flavones (11 downregulated, 15 upregulated), 6 flavonols (2 downregulated, 4 upregulated), 3 flavonoids (1 downregulated, 2 upregulated), 3 flavanones (2 downregulated, 1 upregulated), 6 anthocyanins (3 downregulated, 3 upregulated), 4 isoflavones (0 downregulated, 4 upregulated), and 4 polyphenols (2 downregulated, 2 upregulated). The free radical scavenging ability of flavonoids is proportional to the phenolic hydroxyl groups of the A and B rings. The scavenging ability of phenolic hydroxyl groups on the B ring is stronger than that of the A ring. The position of the 3′ phenolic hydroxyl is the most important (Chen et al., 2002; Bagchi et al., 2014). All the upregulated differential flavonoids metabolites between CP and CK are shown in Table 1. The upregulated flavonoids have abundant phenolic hydroxyl groups in the A and B rings. Some compounds, such as keracyanin, 6‐C‐hexosyl‐luteolin‐5‐O‐pentoside, C‐hexosyl‐luteolin‐5‐O‐feruloylpentoside, and hesperetin, have 3′ phenolic hydroxyl on the B ring.

**Table 1:** A list of upregulated differential flavonoids metabolites between *Pericarpium Citri Reticulatae* (CK) and *Pericarpium Citri Reticulatae* (CP)

No.	Molecular weight (Da)	Compounds	Class	Hydroxyl group and its position
1	2.86E+02	Orobol (5,7,3′,4′‐tetrahydroxyisoflavone)	Isoflavone	5‐OH; 7‐OH; 3′‐OH; 4′‐OH
2	4.30E+02	Formononetin ‐7‐O‐glucoside (Ononin)	Isoflavone	4′‐OH
3	2.86E+02	2′‐Hydroxygenistein	Isoflavone	5‐OH; 7‐OH; 2′‐OH; 4′‐OH
4	2.84E+02	Prunetin	Isoflavone	7‐OH; 4′‐OH
5	5.95E+02	Cyanidin‐ 3‐O‐rutinoside (Keracyanin)	Anthocyanins	4‐OH; 6‐OH; 3′‐OH; 4′‐OH
6	6.11E+02	Cyanidin ‐3,5‐O‐diglucoside (Cyanin)	Anthocyanins	–
7	4.33E+02	Pelargonidin ‐3‐O‐beta‐D‐glucoside	Anthocyanins	5‐OH; 4′‐OH
8	4.64E+02	Isoquercitroside	Flavonoid	4‐OH; 6‐OH; 3′‐OH; 4′‐OH
9	3.44E+02	5,7‐Dihydroxy‐3′,4′,5′‐trimethoxyflavone	Flavonoid	5‐OH; 7‐OH
10	3.14E+02	Velutin	Flavone	7‐OH; 4′‐OH
11	6.24E+02	3′,4′,5′‐Tricetin‐5‐O‐rutinoside	Flavone	3′‐OH; 4′‐OH; 5′‐OH
12	6.24E+02	Chrysoeriol ‐5‐O‐hexosyl‐3‐O‐hexoside	Flavone	7‐OH; 4′‐OH
13	7.28E+02	C‐hexosyl‐apigenin‐5‐O‐hexosyl‐3‐O‐pentoside	Flavone	7‐OH; 4′‐OH
14	7.56E+02	6‐C‐hexosyl‐apigenin‐5‐O‐hexosyl‐6‐O‐hexoside	Flavone	7‐OH; 4′‐OH
15	5.80E+02	6‐C‐hexosyl luteolin‐5‐O‐pentoside	Flavone	7‐OH; 3′‐OH; 4′‐OH
16	5.94E+02	Vitex	Flavone	5‐OH; 7‐OH; 4′‐OH
17	7.56E+02	C‐hexosyl‐luteolin ‐5‐ O‐feruloylpentoside	Flavone	7‐OH; 3′‐OH; 4′‐OH
18	3.02E+02	Hesperetin	Flavone	5‐OH; 7‐OH; 3′‐OH
19	5.64E+02	C‐hexosyl‐apigenin C‐pentoside	Flavone	5‐OH; 7‐OH; 4′‐OH
20	4.76E+02	Chrysoeriol ‐5‐O‐hexoside	Flavone	7‐OH; 4′‐OH
21	6.08E+02	Chrysoeriol ‐7‐O‐rutinoside	Flavone	5‐OH; 4′‐OH
22	2.86E+02	Luteolin	Flavone	4‐OH; 6‐OH; 3′‐OH; 4′‐OH
23	3.02E+02	Tricetin	Flavone	5‐OH; 7‐OH;3′‐OH; 4′‐OH; 5′‐OH
24	5.81E+02	Narirutin	Flavone	7‐OH; 4′‐OH
25	3.02E+02	Ellagic acid	Polyphenol	3‐OH; 4‐OH; 4′‐OH; 5′‐OH
26	4.58E+02	Gallocatechin gallate	Polyphenol	5‐OH; 7‐OH;3′‐OH; 4′‐OH; 5′‐OH
27	4.34E+02	Naringenin	Flavanone	5‐OH; 7‐OH; 4′‐OH
28	3.02E+02	Quercetin	Flavonol	5‐OH; 7‐OH; 3′‐OH; 4′‐OH
29	4.64E+02	Quercetin ‐3‐O‐glucoside (Isotrifoliin)	Flavonol	5‐OH; 7‐OH;3′‐OH; 4′‐OH
30	3.04E+02	Dihydroquercetin (Taxifolin)	Flavonol	5‐OH; 7‐OH;3′‐OH; 4′‐OH

**FIGURE 4:**
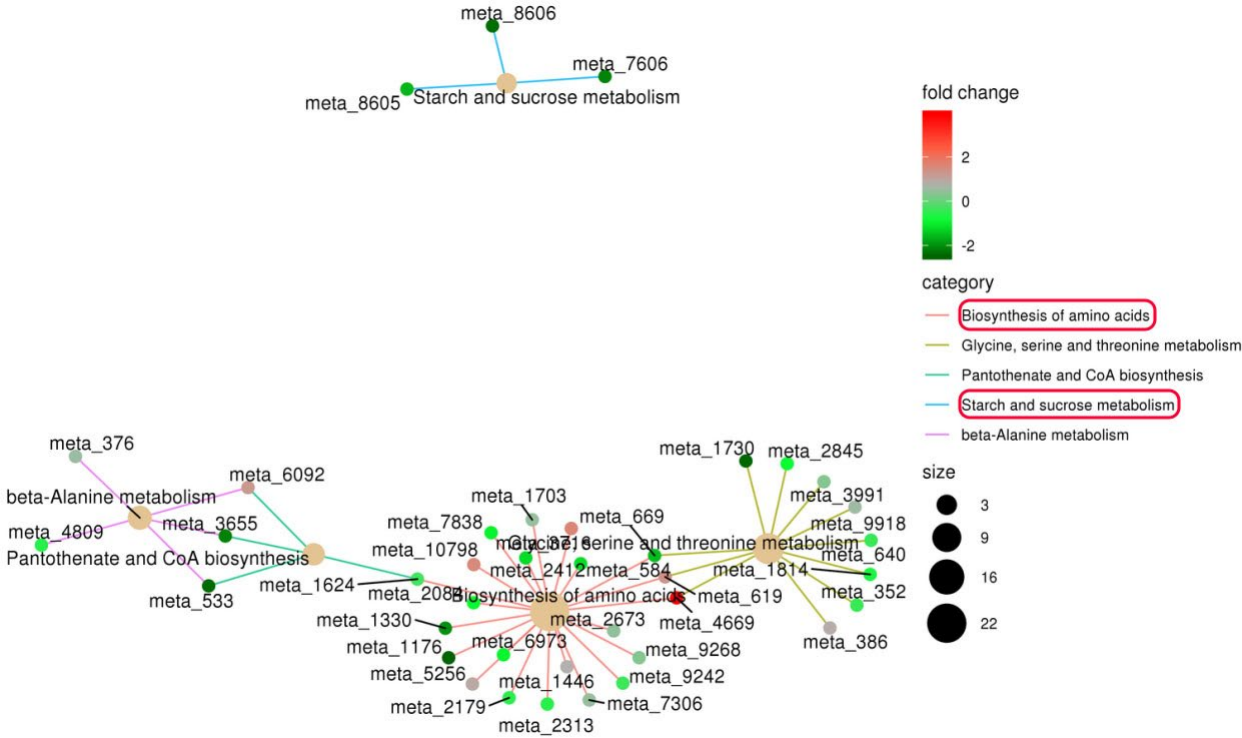
KEGG enrichment network of different metabolites. The light yellow node in the figure is the path, and the small nodes connected to it are the specific metabolites annotated to the path. Color depth indicates the difference multiple takes log_2_ value

In addition, we analyze the enrichment of the differential metabolites and interestingly found that some primary metabolites such as amino acids and sugars changed greatly (Figure 4), which suggest that the mechanism of metabolite transformation by *A. niger* may also be closely related to the changes of energy substances, such as amino acids or sugars.

### Antioxidant and targeted quantitative analysis

#### Antioxidant analysis

**FIGURE 5:**
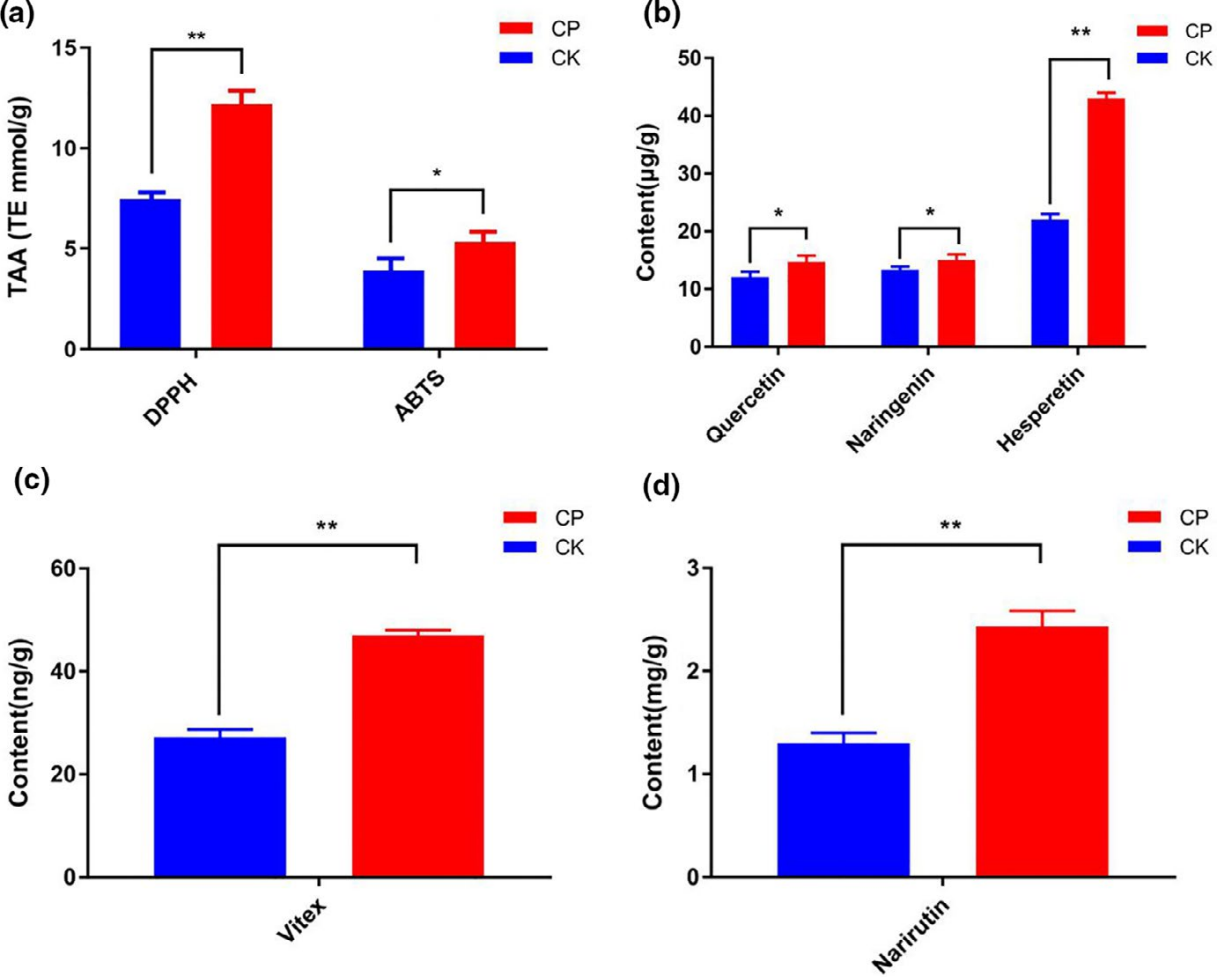
(a) Total antioxidant activity (TAA) in *Pericarpium Citri Reticulatae* (CK) and *Pericarpium Citri Reticulatae* (CP). All values are means ± *SD* (*n* = 6); significant differences were evaluated using one‐way ANOVA. *indicates significant level 0.01 < *p* < .05 and **indicates a significance level *p* < .01. (b) Content of upregulated differential metabolite hesperetin. (c) Content of upregulated differential metabolite vitex. (d) Content of upregulated differential metabolite narirutin

The antioxidant activities of CP and CK were determined by DPPH, ABTS, and FRAP assays. The DPPH, ABTS, and FRAP values of CK were 12.2 ± 1.43 TE mmol/g, 5.34 ± 3.42 TE mmol/g, and 2.63 ± 2.29 FeII mmol/g, respectively, while the DPPH, ABTS, and FRAP values were 7.48 ± 1.52 TE mmol/g, 3.92 ± 2.38 TE mmol/g, and 1.38 ± 1.91 FeII mmol/g, respectively (Table 2). CP exhibited higher antioxidant activity than CK (Figure 5a).

**Table 2:** Results of quercetin, naringenin, hesperetin, vitex, narirutin, DPPH, ABTS, and FRAP free radical‐scavenging activity of CP and CK

	CP	CK	Standard curve
Quercetin^b^ (μg/g)	15.11 ± 3.23	12.11 ± 2.66	*Y* = 0.3385 × 10^2^ *X*−8.6904, *R* ^2^ = .9999
Naringenin^b^ (μg/g)	14.83 ± 2.02	12.33 ± 2.36	*Y* = 0.5845 × 10^7^ *X*−74861.82, *R* ^2^ = .9990
Hesperetin^b^ (μg/g)	47.13 ± 2.11	21.33 ± 2.01	*Y* = 9.2848*X* + 118.85, *R* ^2^ = .9985
Vitex^b^ (ng/g)	48.80 ± 1.47	29.33 ± 1.22	*Y* = 0.1384 × 10^2^ *X* + 381.32, *R* ^2^ = .9975
Narirutin^b^ (mg/g)	2.26 ± 1.33	1.33 ± 1.25	*Y* = 0.2267 × 10^7^ *X*−8907.81, *R^2^* = .9999
DPPH^a^ (TE mM/g)	12.2 ± 1.43	7.48 ± 1.52	*Y* = −0.0007*X* + 0.7451, *R* ^2^ = 0.9999
ABTS^a^ (TE mM/g)	5.34 ± 3.42	3.92 ± 2.38	*Y* = −0.0007*X* + 0.7451, *R* ^2^ = .9999
FRAP^a^ (FeII mM/g)	2.63 ± 2.29	1.38 ± 1.91	*Y* = 3.1202*X*−0.2635, *R* ^2^ = 1

^a^ Data are represented as the mean ± *SD* from six independent experiments (*n* = 6).

^b^ Data are represented as the mean ± *SD* from three independent experiments (*n* = 3).

#### Targeted quantitative analysis

To verify the absolute content of upregulated flavonoids, five upregulated differential flavonoids—quercetin, naringenin, hesperetin, vitex, and narirutin—were quantitatively analyzed by HPLC or LC‐MS. The contents of quercetin, naringenin, hesperetin, vitex, and narirutin in CP were 15.11 ± 3.23 μg/g, 14.83 ± 2.02 μg/g, 47.13 ± 2.11 μg/g, 48.80 ± 1.47 ng/g, and 2.26 ± 1.33 mg/g, respectively (Table 2). The contents of quercetin, naringenin, hesperetin, vitex, and narirutin in CK were 12.11 ± 2.66 μg/g, 12.33 ± 2.36 μg/g, 21.33 ± 2.01 μg/g, 29.33 ± 1.22 ng/g, and 1.33 ± 1.25 mg/g, respectively. CP exhibited higher contents than CK (Figure 5b‐d). The absolute quantitative results were consistent with the relative quantitative results. Previous studies have reported a linear positive correlation between flavonoid compounds rich in phenolic hydroxyl groups with their antioxidant capacities (Wang et al., 2017). Therefore, microbial transformation of PCR by *A. niger* can change the composition of metabolites, transform flavonoids, and promote the accumulation of phenolic hydroxyl compounds. The higher antioxidant activity of CP may be attributed to the presence of a higher level of flavonoids with rich phenolic hydroxyl groups.

## DISCUSSION

Bioconversion technology has attracted attention because of its potential for producing novel active chemicals (Hidalgo et al., 2018). *A. niger* is a eukaryote that is considered safe in biomicrobial transformation (Schuster et al., 2002). The metabolites of *A. niger* contain abundant metabolic enzymes, which can efficiently transform different types of compounds (Cao et al., 2015; Kumar & Pandey, 2013). *A. niger* transformation of monomer compounds has been a focus because of its great potential for producing novel active components. However, the conversion of complex mixtures has rarely been reported. The purpose of this study is to provide a practical method for the additional development and use of PCR using microbial transformation.

Previously, the UPLC‐ESI‐MS/MS‐based widely targeted metabolomics has been widely used in the differential analysis of samples (Wang et al., 2019). HPLC and LC‐MS methods have also been used to quantitative analysis of sample compositions (Suresh et al., 2018). In this study, microbial biotransformation of PCR by *A. niger* was analyzed based on the combination of UPLC‐ESI‐MS/MS, HPLC, and LC‐MS methods, and the antioxidant activity was also compared using DPPH, FRAP, and ABTS methods. The results demonstrated detection of 2,136 metabolites among which 767 metabolites were identified in CP and CK. Heat‐map analysis, principal component analysis (PCA), and orthogonal signal correction and partial least squares‐discriminant analysis (OPLS‐DA) were used to clearly discriminate between CP and CK. The results revealed that *A. niger* participated in the transformation of metabolites and had a great impact on the metabolism of PCR. The changes of these metabolites are often closely related to the corresponding changes in biological activity. Ma et al. reported that the biological activity of plant extracts were strongly positively correlated with their changes in active ingredients after microbial biotransformation (Ma et al., 2019).

To understand the difference of antioxidant activity between CP and CK, 109 differential flavonoids metabolites were screened out and the number of upregulated and downregulated differential flavonoids was analyzed. The number of upregulated flavonoids was significantly greater than the downregulated flavonoids, and the upregulated flavonoids contained abundant polyhydroxy groups. In general, flavonoids often contain polyhydroxy groups which serve as antioxidants due to their free radical scavenging activity (Gil‐Cardoso et al., 2016). In particular, 3′ phenolic hydroxyl on the B ring can greatly increase its ability to scavenge free radicals. We concluded that the metabolic transformation produced by *A. niger* can significantly improve the antioxidant activity of PCR. To verify these results, three different methods, DPPH, FRAP, and ABTS, were used to compare the antioxidant activity between CK and CP. The results demonstrated that CP exhibited higher antioxidant activity than CK. The increased antioxidant activity of PCR transformed by *A. niger* is closely related to the enrichment of polyhydroxyflavones. A study on microbial biotransformation also revealed that the plant extract present higher antioxidant activity after *A. niger* fermentation and believed that it could be attributed to an increase in the concentration of original active compounds (Wu, Chen, et al., 2018). Other previous reports also have suggested that *A. niger* has the ability to produce different bioactive compounds by different metabolic pathways, and it can also increase the physiological and biochemical activities of biological substrates by modifying their original molecules. Such as *A. niger* N402 was reported has steroid‐hydroxylating activity and can introduce a hydroxyl group into the progesterone molecule (Savinova, et al., 2018). Another *A. niger* JMU‐TS528 could transform rutin to isoquercitrin (Li et al., 2020). *Aspergillus niger* NRRL 3,122 could promote the hydrolysis of soy flour isoflavone glycosides to their aglycone. All these results are attributed to the specific enzymes produced by *A. niger* (Abdella et al., 2018).

## CONCLUSION

We analyzed the changes of metabolites before (CK) and after (CP) microbial transformation of *Pericarpium Citri Reticulatae* (PCR) by *Aspergillus niger* using UPLC‐ESI‐MS/MS, HPLC, and LC‐MS methods, and we compared the antioxidant activity using three different methods (DPPH, FRAP, and ABTS). Qualitative and quantitative analyses showed that microbial transformation of PCR by *A. niger* could transform flavonoids and also promote the accumulation of phenolic hydroxyl compounds. The antioxidant assay demonstrated that CP had significantly higher free radical‐scavenging activity than CK, and the higher antioxidant activity of CP may be attributed to the presence of a high level of flavonoids with rich phenolic hydroxyl groups. These results provide useful information related to the mechanism of flavonoid transformation by *A. niger*. The strain of *A. niger* may also have a great influence on the high efficiency extraction of flavonoids, the conversion of active ingredients, and the improvement of PCR quality.

## CONFLICT OF INTEREST

All the authors declare that there are no conflicts of interest.

## ETHICAL APPROVAL

This study does not involve any human or animal testing.

## Supporting information

Tab S1

Tab S2

Tab S3

## References

[fsn32049-bib-0001] Abdella, A. , El‐Baz, A. F. , Ibrahim, I. A. , Mahrous, E. E. , & Yang, S. T. (2018). Biotransformation of soy flour isoflavones by Aspergillus niger NRRL 3122 β‐glucosidase enzyme. Natural Product Research, 32, 2382–2391. 29224366 10.1080/14786419.2017.1413569

[fsn32049-bib-0002] Ardila, J. A. , Funari, C. S. , Andrade, A. M. , Cavalheiro, A. J. , & Carneiro, R. L. (2015). Cluster analysis of commercial samples of Bauhinia spp. using HPLC‐UV/PDA and MCR‐ALS/PCA without peak alignment procedure. Phytochemical Analysis, 26, 367–373. 26047147 10.1002/pca.2571

[fsn32049-bib-0003] Bagchi, D. , Swaroop, A. , Preuss, H. G. , & Bagchi, M. (2014). Free radical scavenging, antioxidant and cancer chemoprevention by grape seed proanthocyanidin: An overview. Mutation Research, 768, 69–73. 10.1016/j.mrfmmm.2014.04.004 24751946

[fsn32049-bib-0004] Bao, L. , Hu, L. , Zhang, Y. , & Wang, Y. I. (2016). Hypolipidemic effects of flavonoids extracted from *Lomatogonium rotatum* . Experimental and Therapeutic Medicine, 11, 1417–1424. 10.3892/etm.2016.3038 27073459 PMC4812540

[fsn32049-bib-0005] Basu, A. , Das, A. S. , Majumder, M. , & Mukhopadhyay, R. (2016). Antiatherogenic roles of dietary flavonoids chrysin, quercetin, and luteolin. Journal of Cardiovascular Pharmacology, 68, 89–96. 10.1097/FJC.0000000000000380 27385185

[fsn32049-bib-0006] Bianchini, L. F. , Arruda, M. F. , Vieira, S. R. , Campelo, P. M. , Grégio, A. M. , & Rosa, E. A. (2015). Microbial biotransformation to obtain new antifungals. Frontiers in Microbiology, 6, 1433 10.3389/fmicb.2015.01433 26733974 PMC4689855

[fsn32049-bib-0007] Cao, H. , Chen, X. , Jassbi, A. R. , & Xiao, J. (2015). Microbial biotransformation of bioactive flavonoids. Biotechnology Advances, 33, 214–223. 10.1016/j.biotechadv.2014.10.012 25447420

[fsn32049-bib-0008] Caspani, G. , Kennedy, S. , Foster, J. A. , & Swann, J. (2019). Gut microbial metabolites in depression: Understanding the biochemical mechanisms. Microbial Cell, 6, 454–481. 10.15698/mic2019.10.693 31646148 PMC6780009

[fsn32049-bib-0009] Chen, C. , Li, S. X. , Wang, S. M. , & Liang, S. W. (2012). Investigation into the anti‐thrombosis effect and contents of total saponins and flavonoids in the bioactive fraction of Naodesheng prescription. Journal of Ethnopharmacology, 144, 208–212. 10.1016/j.jep.2012.09.007 22982395

[fsn32049-bib-0010] Chen, J. W. , Zhu, Z. Q. , Hu, T. X. , & Zhu, D. Y. (2002). Structure‐activity relationship of natural flavonoids in hydroxyl radical‐scavenging effects. Acta Pharmacologica Sinica, 23, 667–672. 12100765

[fsn32049-bib-0011] Das, S. , & Rosazza, J. P. (2006). Microbial and enzymatic transformations of flavonoids. Journal of Natural Products, 69, 499–508. 16562863 10.1021/np0504659

[fsn32049-bib-0012] Gil‐Cardoso, K. , Ginés, I. , Pinent, M. , Ardévol, A. , Blay, M. , & Terra, X. (2016). Effects of flavonoids on intestinal inflammation, barrier integrity and changes in gut microbiota during diet‐induced obesity. Nutrition Research Reviews, 29, 234–248. 10.1017/S0954422416000159 27841104

[fsn32049-bib-0013] Gonzales, G. B. , Smagghe, G. , Grootaert, C. , Zotti, M. , Raes, K. , & Van Camp, J. (2015). Flavonoid interactions during digestion, absorption, distribution and metabolism: A sequential structure‐activity/property relationship‐based approach in the study of bioavailability and bioactivity. Drug Metabolism Reviews, 47, 175–190. 10.3109/03602532.2014.1003649 25633078

[fsn32049-bib-0014] Hidalgo, D. , Sanchez, R. , Lalaleo, L. , Bonfill, M. , Corchete, P. , & Palazon, J. (2018). Biotechnological production of pharmaceuticals and biopharmaceuticals in plant cell and organ cultures. Current Medicinal Chemistry, 25, 3577–3596. 10.2174/0929867325666180309124317 29521202

[fsn32049-bib-0015] Jia, L. G. , Sheng, Z. W. , Xu, W. F. , Li, Y. X. , Liu, Y. G. , Xia, Y. J. , & Zhang, J. H. (2012). Modulation of anti‐oxidation ability by proanthocyanidins during germination of Arabidopsis thaliana seeds. Molecular Plant, 5, 472–481. 10.1093/mp/ssr089 22115918

[fsn32049-bib-0016] Jiang, J. , Yan, L. , Shi, Z. , Wang, L. , Shan, L. , & Efferth, T. (2019). Hepatoprotective and anti‐inflammatory effects of total flavonoids of Qu Zhi Ke (peel of Citrus changshan‐huyou) on non‐alcoholic fatty liver disease in rats via modulation of NF‐κB and MAPKs. Phytomedicine, 64, 153082 10.1016/j.phymed.2019.153082 31541796

[fsn32049-bib-0017] Jiang, M. , Zhu, M. , Wang, L. , & Yu, S. (2019). Anti‐tumor effects and associated molecular mechanisms of myricetin. Biomedicine & Pharmacotherapy, 120, 109506 10.1016/j.biopha.2019.109506 31586904

[fsn32049-bib-0018] Kang, X. , Csetenyi, L. , & Gadd, G. M. (2019). Biotransformation of lanthanum by Aspergillus niger. Applied Microbiology and Biotechnology, 103, 981–993. 10.1007/s00253-018-9489-0 30443797 PMC6373195

[fsn32049-bib-0019] Kostrzewa‐Susłow, E. , Dymarska, M. , & Janeczko, T. (2014). Microbial transformations of 3‐methoxyflavone by strains of Aspergillus niger. Polish Journal of Microbiology, 63, 111–114. 25033671

[fsn32049-bib-0020] Kostrzewa‐Susłow, E. , & Janeczko, T. (2012). Microbial transformations of 7‐hydroxyflavanone. Scientific World Journal, 2012, 254929 10.1100/2012/254929 22654578 PMC3361144

[fsn32049-bib-0021] Kumar, S. , & Pandey, A. K. (2013). Chemistry and biological activities of flavonoids: An overview. Scientific World Journal, 29(2013), 162750 10.1155/2013/162750 PMC3891543 24470791

[fsn32049-bib-0022] Li, L. J. , Liu, X. Q. , Du, X. P. , Wu, L. , Jiang, Z. D. , Ni, H. , Li, Q. B. , & Chen, F. (2020). Preparation of isoquercitrin by biotransformation of rutin using α‐L‐rhamnosidase from Aspergillus niger JMU‐TS528 and HSCCC purification. Preparative Biochemistry & Biotechnology, 50, 1–9. 31441715 10.1080/10826068.2019.1655763

[fsn32049-bib-0023] Ma, R. J. , Yang, L. , Bai, X. , Li, J. Y. , Yuan, M. Y. , Wang, Y. Q. , Xie, Y. , Hu, J. M. , & Zhou, J. (2019). Phenolic constituents with antioxidative, tyrosinase inhibitory and anti‐aging activities from dendrobium loddigesii rolfe. Natural Products and Bioprospecting, 9, 329–336. 10.1007/s13659-019-00219-y 31630376 PMC6814690

[fsn32049-bib-0024] Mahmoud, Y. A. , Assawah, S. W. , El‐Sharkawy, S. H. , & Abdel‐Salam, A. (2008). Flavone biotransformation by Aspergillus niger and the characterization of two newly formed metabolites. Mycobiology, 36, 121–133. 23990746 10.4489/MYCO.2008.36.2.121 PMC3755235

[fsn32049-bib-0025] Miura, M. , Nogami, M. , Sakai, M. , Sato, M. , & Yatsushiro, T. (2020). Rapid LC‐MS/MS determination of hesperidin in fermented tea prepared from unripe Satsuma Mandarin (Citrus unshiu) fruits and third‐crop green tea (Camellia sinensis) leaves. Analytical Sciences, 36, 1243–1249. 32475895 10.2116/analsci.20P100

[fsn32049-bib-0026] Mozaffari, H. , Daneshzad, E. , Surkan, P. J. , & Azadbakht, L. (2018). Dietary total antioxidant capacity and cardiovascular disease risk factors: A systematic review of observational studies. Journal of the American College of Nutrition, 37, 533–545. 10.1080/07315724.2018.1441079 29714643

[fsn32049-bib-0027] Okuno, Y. , & Miyazawa, M. (2004). Biotransformation of nobiletin by Aspergillus niger and the antimutagenic activity of a metabolite, 4'‐hydroxy‐5,6,7,8,3'‐pentamethoxyflavone. Journal of Natural Products, 67, 1876–1878. 15568779 10.1021/np034007g

[fsn32049-bib-0028] Parshikov, I. A. , & Sutherland, J. B. (2015). Biotransformation of Steroids and Flavonoids by Cultures of Aspergillus niger. Applied Biochemistry and Biotechnology, 176, 903–923. 10.1007/s12010-015-1619-x 25951777

[fsn32049-bib-0029] Polak, J. , & Bartoszek, M. (2018). A new equation for converting the parameter EC50 into the total antioxidant capacity TEAC and vice versa. Food Chemistry, 248, 46–51. 10.1016/j.foodchem.2017.12.030 29329869

[fsn32049-bib-0030] Sanchez‐Gonzalez, M. , & Rosazza, J. P. (2004). Microbial transformations of chalcones: Hydroxylation, O‐demethylation, and cyclization to flavanones. Journal of Natural Products, 67, 553–558. 15104483 10.1021/np030448o

[fsn32049-bib-0031] Savinova, O. S. , Solyev, P. N. , Vasina, D. V. , Tyazhelova, T. V. , Fedorova, T. V. , & Savinova, T. S. (2018). Biotransformation of progesterone by the ascomycete Aspergillus niger N402. Biochemistry (Moscow), 83, 26–31. 10.1134/S0006297918010030 29534665

[fsn32049-bib-0032] Schuster, E. , Dunn‐Coleman, N. , Frisvad, J. C. , & VanDijck, P. W. (2002). On the safety of Aspergillus niger–a review. Applied Microbiology and Biotechnology, 59, 426–435. 10.1007/s00253-002-1032-6 12172605

[fsn32049-bib-0033] Shahinozzaman, M. , Taira, N. , Ishii, T. , Halim, M. A. , Hossain, M. A. , & Tawata, S. (2018). Anti‐inflammatory, anti‐diabetic, and anti‐Alzheimer's effects of prenylated flavonoids from Okinawa Propolis: an investigation by experimental and computational studies. Molecules, 23, 2479 10.3390/molecules23102479 PMC6222853 30262742

[fsn32049-bib-0034] Shimizu, T. , Watanabe, M. , Fernie, A. R. , & Tohge, T. (2018). Targeted LC‐MS analysis for plant secondary metabolites. Methods in Molecular Biology, 1778, 171–181. 29761438 10.1007/978-1-4939-7819-9_12

[fsn32049-bib-0035] Sirivibulkovit, K. , Nouanthavong, S. , & Sameenoi, Y. (2018). Paper‐based DPPH assay for antioxidant activity analysis. Analytical Sciences, 34, 795–800. 10.2116/analsci.18P014 29998961

[fsn32049-bib-0036] Stankov‐Jovanović, V. P. , Ilić, M. D. , Mitić, V. D. , Mihajilov‐Krstev, T. M. , Simonović, S. R. , Mandić, S. D. , Tabet, J. C. , & Cole, R. B. (2015). Secondary metabolites of Seseli rigidum: Chemical composition plus antioxidant, antimicrobial and cholinesterase inhibition activity. Journal of Pharmaceutical and Biomedical Analysis, 111, 78–90. 10.1016/j.jpba.2015.03.015 25863020

[fsn32049-bib-0037] Suresh, P. S. , Srinivas, N. R. , & Mullangi, R. (2018). Review of HPLC and LC‐MS/MS assays for the determination of various nonsteroidal anti‐androgens used in the treatment of prostate cancer. Biomedical Chromatography, 32(1). 10.1002/bmc.4034 28636139

[fsn32049-bib-0038] Tao, L. , Qu, X. , Zhang, Y. , Song, Y. , & Zhang, S. X. (2019). Prophylactic therapy of Silymarin (Milk Thistle) on antituberculosis drug‐induced liver injury: A meta‐analysis of randomized controlled trials. Canadian Journal of Gastroenterology and Hepatology, 2019, 3192351 10.1155/2019/3192351 30733935 PMC6348824

[fsn32049-bib-0039] Triba, M. N. , Moyec, L. , Amathieu, R. , Goossens, C. , Bouchemal, N. , Nahon, P. , Rutledge, D. N. , & Savarin, P. (2015). PLS/OPLS models in metabolomics: The impact of permutation of dataset rows on the K‐fold cross‐validation quality parameters. Molecular BioSystems, 11, 13–19. 10.1039/C4MB00414K 25382277

[fsn32049-bib-0040] Vazhappilly, C. G. , Ansari, S. A. , Al‐Jaleeli, R. , Al‐Azawi, A. M. , Ramadan, W. S. , Menon, V. , Hodeify, R. , Siddiqui, S. S. , Merheb, M. , Matar, R. , & Radhakrishnan, R. (2019). Role of flavonoids in thrombotic, cardiovascular, and inflammatory diseases. Inflammopharmacology, 27, 863–869. 10.1007/s10787-019-00612-6 31309484

[fsn32049-bib-0041] Wang, F. , Chen, L. , Chen, H. , Chen, S. , & Liu, Y. (2019). Analysis of Flavonoid Metabolites in Citrus Peels (Citrus reticulata "Dahongpao") Using UPLC‐ESI‐MS/MS. Molecules, 24, 2680 10.3390/molecules24152680 PMC6696472 31344795

[fsn32049-bib-0042] Wang, F. , Chen, L. , Li, F. Q. , Liu, S. J. , Chen, H. P. , & Liu, Y. P. (2018). The increase of flavonoids in Pericarpium Citri Reticulatae (PCR) induced by fungi promotes the increase of antioxidant activity. Evidence‐Based Complementary and Alternative Medicine: Ecam, 2018, 2506037 10.1155/2018/2506037 30622593 PMC6304648

[fsn32049-bib-0043] Wang, Y. , Qian, J. , Cao, J. , Wang, D. , Liu, C. , Yang, R. , Li, X. , & Sun, C. (2017). Antioxidant capacity, anticancer ability and flavonoids composition of 35 citrus (Citrus reticulata Blanco) varieties. Molecules, 22, 1114 10.3390/molecules22071114 PMC6152254 28678176

[fsn32049-bib-0044] Wu, L. , Chen, C. , Cheng, C. , Dai, H. , Ai, Y. , Lin, C. , & Chung, Y. (2018). Evaluation of tyrosinase inhibitory, antioxidant, antimicrobial, and antiaging activities of magnolia officinalis extracts after Aspergillus niger fermentation. BioMed Research International, 15(2018), 5201786. 10.1155/2018/5201786 PMC6276509 30581856

[fsn32049-bib-0045] Wu, W. , Jiao, C. , Li, H. , Ma, Y. , Jiao, L. , & Liu, S. (2018). LC‐MS based metabolic and metabonomic studies of Panax ginseng. Phytochemical Analysis, 29, 331–340. 29460310 10.1002/pca.2752

[fsn32049-bib-0046] Xiao, J. , Xiao, S. , Xu, Z. , Luo, J. , Lu, M. X. , Lu, M. F. , & Liu, X. Q. (2020). Simultaneous determination of 10 flavonoids in 16 species of Acanthopanax by HPLC. Traditional Chinese Medicine, 9, 2181–2188. 10.13863/j.issn1001-4454.2020.09.021

[fsn32049-bib-0047] Xie, Y. , Yang, W. , Tang, F. , Chen, X. , & Ren, L. (2015). Antibacterial activities of flavonoids: Structure‐activity relationship and mechanism. Current Medicinal Chemistry, 22, 132–149. 25245513 10.2174/0929867321666140916113443

[fsn32049-bib-0048] Xu, J. , Yang, L. , Zhao, S. J. , Wang, Z. T. , & Hu, Z. B. (2012). An efficient way from naringenin to carthamidine and isocarthamidine by Aspergillus niger. World Journal of Microbiology & Biotechnology, 28, 1803–1806. 10.1007/s11274-011-0934-9 22805963

[fsn32049-bib-0049] You, H. J. , Ahn, H. J. , & Ji, G. E. (2010). Transformation of rutin to antiproliferative quercetin‐3‐glucoside by Aspergillus niger. Journal of Agriculture and Food Chemistry, 58, 10886–10892. 10.1021/jf102871g 20886886

[fsn32049-bib-0050] Yu, X. , Sun, S. , Guo, Y. , Liu, Y. , Yang, D. , Li, G. , & Lü, S. (2018). Citri Reticulatae Pericarpium (Chenpi): Botany, ethnopharmacology, phytochemistry, and pharmacology of a frequently used traditional Chinese medicine. Journal of Ethnopharmacology, 28, 265–282. 10.1016/j.jep.2018.03.031 29628291

[fsn32049-bib-0051] Yu, X. , Zhang, Y. , Wang, D. , Jiang, L. , & Xu, X. (2018). Identification of three kinds of *Citri Reticulatae Pericarpium* based on deoxyribonucleic acid barcoding and high‐performance liquid chromatography‐diode array Detection‐Electrospray Ionization/Mass Spectrometry/Mass Spectrometry combined with Chemometric analysis. Pharmacogn Mag, 4, 64–69. 10.4103/pm.pm_581_16 PMC5858244 29576703

[fsn32049-bib-0052] Zakaryan, H. , Arabyan, E. , Oo, A. , & Zandi, K. (2017). Flavonoids: Promising natural compounds against viral infections. Archives of Virology, 162, 2539–2551. 10.1007/s00705-017-3417-y 28547385 PMC7087220

